# TLR 9 Activation in Dendritic Cells Enhances Salmonella Killing and Antigen Presentation via Involvement of the Reactive Oxygen Species

**DOI:** 10.1371/journal.pone.0013772

**Published:** 2010-10-29

**Authors:** Amit Lahiri, Ayan Lahiri, Priyanka Das, Janakiraman Vani, M. S. Shaila, Dipshikha Chakravortty

**Affiliations:** Center for Infectious Disease Research and Biosafety Laboratories, Department of Microbiology and Cell Biology, Indian Institute of Science, Bangalore, India; CNRS - Université Aix-Marseille, France

## Abstract

Synthetic CpG containing oligodeoxynucleotide Toll like receptor-9 agonist (CpG DNA) activates innate immunity and can stimulate antigen presentation against numerous intracellular pathogens. It was observed that *Salmonella* Typhimurium growth can be inhibited by the CpG DNA treatment in the murine dendritic cells. This inhibitory effect was mediated by an increased reactive oxygen species production. In addition, it was noted that CpG DNA treatment of dendritic cells during *Salmonella* infection leads to an increased antigen presentation. Further this increased antigen presentation was dependent on the enhanced reactive oxygen species production elicited by Toll like receptor-9 activation. With the help of an exogenous antigen it was shown that *Salmonella* antigen could also be cross-presented in a better way by CpG induction. These data collectively indicate that CpG DNA enhance the ability of murine dendritic cells to contain the growth of virulent *Salmonella* through reactive oxygen species dependent killing.

## Introduction

Dendritic cells (DCs) are specialized antigen presenting cells which constantly screen the environment for foreign particles [1] and engulf them via a variety of ways like phagocytosis, macropinocytosis, caveolin-mediated or clathrin-dependent endocytosis [2], [3]. DCs function at the dividing line of innate and adaptive immunity [4] and regulate the T cell response [5], [6]. DCs capture antigens in the peripheral tissues and present the processed antigen via the major histocompatibility complex (MHC) I and II receptors [7], [8]. *Salmonella* in its turn is a very successful pathogen. It is a facultative intracellular pathogen and resides in macrophages and DCs by virtue of its pathogenicity island encoded virulence factors which are required for intracellular survival, replication and for the efficient colonization of deeper tissues. *Salmonella* is capable of causing symptoms ranging from self limiting diarrhea and localized gastrointestinal inflammation to the systemic typhoid fever. The mice model of infection mimics the pathogenesis of human typhoid fever.

Toll like receptors (TLRs) are germ line receptors expressed on DCs and recognize infectious agents through the various moieties present on them and act as a bridge between innate and adaptive immunity [9]. Their localization is determined by the nature of the ligand that they bind to. For instance, Toll like receptor-9 (TLR-9) is localized in the late endosomes or lysosomes, where it detects unmethylated CpG motifs in double stranded DNA. Ligand receptor engagement leads to the docking of adaptor molecules like MyD88 to TLRs and recruitment of proteins belonging to the IRAK family [10], [11]. This ultimately leads to the NFκb activation and gene expression for the production of inflammatory cytokines like IL-6, IL-12 and TNF-α which lead to further recruitment of successive waves of immature DCs and monocytes to the portals of pathogen entry [12]. Therefore, TLR activation helps in mounting a more prominent T cell response, better killing of the pathogen and thus utilizing TLR signaling could be an effective strategy to clear the invading pathogen. The same has been shown in cultured hepatocytes where CpG treatment led to an increased TLR-9 expression and peroxide formation resulting in an enhanced killing of *Salmonella* [13].

Peroxide in a cell is formed by the NADPH oxidase, a multicomponent enzyme system responsible for releasing reactive oxygen species (ROS) in a process known as the respiratory burst [14], [15]. A voluminous literature exists pertaining to the role of ROS in regulating antigen presentation. ROS mediated maintenance of the intracellular redox potential of macrophages and DCs is crucial for their antigen presenting capability. In addition, it is also reported that antigen specific bidirectional dendritic cell and T cell interaction is blocked by inhibiting NADPH oxidase due to inhibition of ROS productio. In addition, published data suggest that phagocyte NADPH oxidase prevents acidification of the phagosomes and thereby promote antigen cross presentation [6], [16]–[21]. Thus, it can be clearly concluded that by modulating the ROS profile of an infected host, its antigen presentation capacity can be altered.

In this study, we sought to examine whether CpG-DNA treatment can bring about the growth attenuation of *Salmonella* in DCs and to identify the mechanisms involved in the antibacterial response of CpG-DNA treated DCs. We further hypothesized that CpG induced ROS can enhance the antigen presentation capacity of the *Salmonella* infected DCs and can therefore alter the fate of the infection.

## Results

### TLR-9 activation decrease *Salmonella* burden in DCs

**Figure 1:**
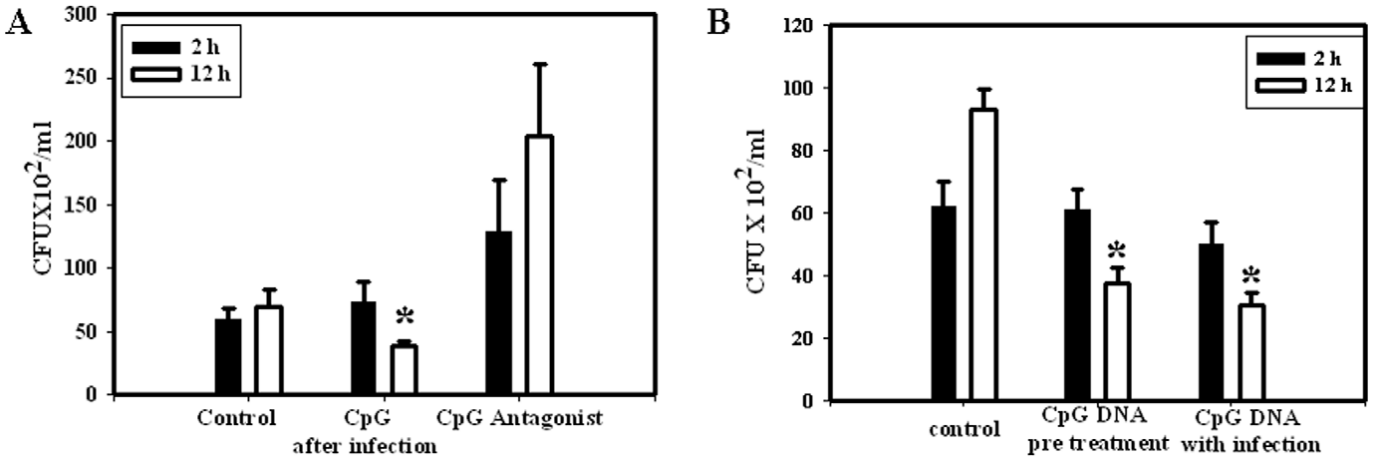
CpG DNA treatment leads to *Salmonella* growth attenuation in DCs. DCs were infected with the WT *Salmonella* at a MOI of 10. After 25 min of infection, cells were treated with TLR-9 ligand or antagonist along with 10 µg/ml gentamicin (A). The CpG treatment was done 12 h before infection or along with infection (B). At 2 and 12 h post infection, macrophages were lysed by the addition of 0.1% Triton X-100, and serial dilutions were plated on LB agar for the enumeration of intracellular bacteria. The intracellular CFU was determined for both time points, and the rates of intracellular replication were expressed as CFU/ml. Experiments were performed in triplicate on three different occasions and the standard errors from the means are shown. Statistical significance was defined as follows: * *P*<0.05 (Student's *t* test).

To understand the role of TLR-9 in *Salmonella* infection, we infected dendritic cells with *Salmonella* in the presence of the TLR-9 ligand, CpG DNA. Previous report suggests that CpG DNA can change the conformation of TLR-9 and activate the downstream signaling [22]. In accordance with previous reports, we also found that in the untreated DCs, *Salmonella* load from 2 to 12 h of infection remained static. However, the data presented in Fig. 1A clearly indicate that the CpG DNA mediated activation of the TLR-9 post infection led to a significantly diminished *Salmonella* burden in DCs. The essential role of TLR-9 was further supported by the observation that the significantly reduced proliferation of *Salmonella* was not observed in the DCs wherein the TLR-9 signaling was inhibited by a specific TLR-9 antagonist. The antagonist treatment does not significantly increase the bacterial load even at 2 or 12 h of growth indicating *Salmonella* infection does not itself activate TLR-9 signaling. We further measured *Salmonella* proliferation in TLR-9 ligand pre-treated cells and also when TLR-9 ligand induction was performed along with the infection. In both conditions we observed a diminished burden as shown in Fig. 1B indicating that TLR-9 treatment at pre, post or along with the *Salmonella* infection leads to an enhanced killing of the bacteria. The ligand and the antagonist treatment do not lead to the killing of the dendritic cells. No decrease in cell number or viability was observed in any of the experimental conditions as indicated by MTT assay (Figure S1). When we checked for the bacterial invasion in the treated cells, there was no difference in the uptake upon ligand or antagonist treatment (data not shown).

### TLR-9 protein level is enhanced upon CpG treatment

**Figure 2:**
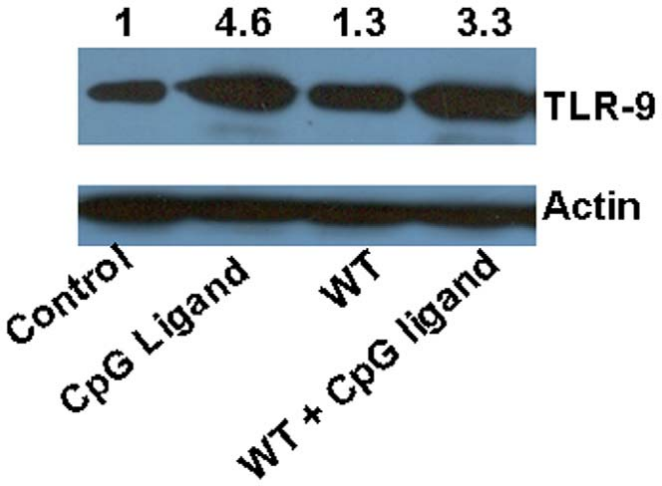
CpG treatment increases the TLR-9 protein level in the *Salmonella* infected DCs. The total protein after 12 h of infection or treatment was extracted from the cell and 100 µg of the total protein was loaded on SDS page and subjected to Western blot analysis to check the amount of TLR-9 and β-actin. Blot is representative of three independent experiments. Numbers in parenthesis are the values obtained after densitometric image analysis in which density values of TLR-9 were normalized to those of β-actin.

To determine the effect of *Salmonella* infection or the CpG ligand treatment on TLR-9 expression, we performed western blot to determine the protein level of TLR-9 in DCs. CpG treatment has earlier been reported to increase the transcription of TLR-9 [23]. Further, it has been shown that DNA isolated from pathogenic *Salmonella* also leads to the upregulation of the TLR-9 protein expression [24]. Interestingly, in the same study it was observed that live *Salmonella* infection did not increase TLR-9 expression as the bacterial DNA might not have been accessible to the TLR-9 receptor. The data shown in Fig. 2 represent TLR9 expression. In accordance with previous reports; we also observed that *Salmonella* infection does not significantly increase the TLR-9 level in the infected DCs compared to the control cells. However, with the cognate ligand treatment alone, there was a 4-fold increase in the TLR-9. Also in the case of *Salmonella* infection along with the CpG treatment there was a significant increase in the TLR-9 level. These results clearly prove that CpG treatment allows *Salmonella* infected DCs to induce an enhanced level of TLR-9 expression, thus enabling a better clearance of the pathogen.

### The mechanism of TLR-9 mediated anti *Salmonella* response

**Figure 3:**
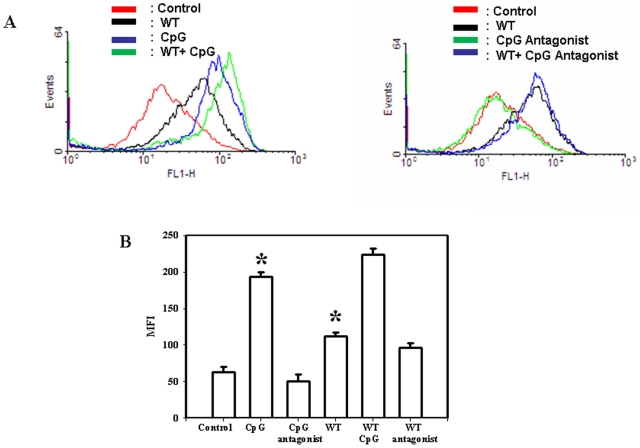
CpG DNA treatment enhance the ROS production in *Salmonella* infected DCs. (A). The cells under various conditions were incubated with 10 µM DCFDA and were analysed by FACS for ROS production. In the left panel, the uninfected cells (red) and the infected cells (black) without the CpG DNA treatment are compared with the uninfected cells (blue) and infected cells (green) treated with the CpG DNA. In the right panel, the uninfected cells (red) and the infected cells (black) without the CpG antagonist treatment are compared with the uninfected cells (green) and infected cells (blue) treated with the CpG antagonist (B). Mean Fluorescence Intensity (MFI) of the samples are plotted from all the above mentioned experimental conditions. Experiments were performed in triplicate on three different occasions and the standard errors from the means are shown. Statistical significance was defined as follows: * *P*<0.05. (Student's *t* test).

**Figure 4:**
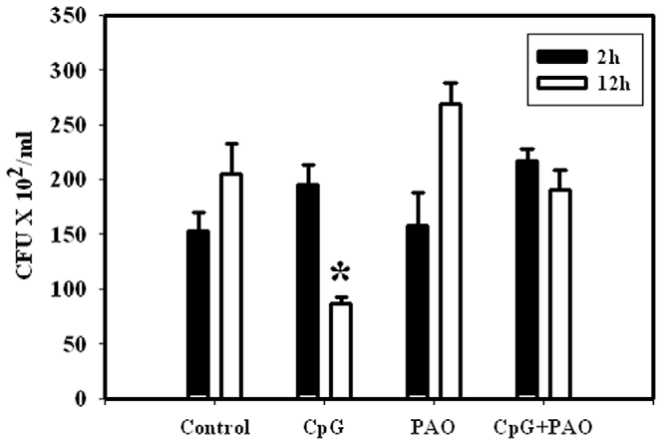
ROS inhibition by PAO rescued the *Salmonella* proliferation defect in the CpG DNA treated DCs. DCs were pretreated for 30 min with or without PAO (5 µM, phenylarsine oxide) and then infected with the WT *Salmonella* with or without 10 µg/ml CpG DNA. After 2 h (open bar) and 16 h (filled bar) of infection the intracellular CFU was determined for both time points, and the rates of intracellular replication were expressed as CFU/ml. Experiments were performed in triplicate on two different occasions and the standard errors from the means are shown. Statistical significance was defined as follows: * *P*<0.05. (Student's *t* test).

In order to better understand the mechanism of CpG mediated *Salmonella* growth attenuation, we went ahead to measure the amount of ROS and nitric oxide (NO) production in the CpG treated DCs. Our results showed that there was no difference in the NO production between CpG treated and untreated *Salmonella* infected DCs (data not shown). We next went ahead to check whether the CpG induced antibacterial activity could be due to an enhanced ROS production. In accordance with our hypothesis, we indeed observed an enhanced ROS production in the infected DCs after CpG treatment (Fig. 3A). In the infected cells treated with CpG, the MFI was 2-fold more than the untreated cells for the DAF2DA staining (Fig. 3B). The TLR-9 antagonist treatment showed a reduced ROS production. To further evaluate our result, we next blocked the ROS production in the CpG treated DCs and examined *Salmonella* proliferation. Data presented in Fig. 4 indicate that the *Salmonella* attenuation with CpG treatment is no longer observed in presence of the ROS inhibitor PAO. Thus, our results clearly suggest that CpG mediated killing of *Salmonella* is mediated by an enhanced ROS production.

**Figure 5:**
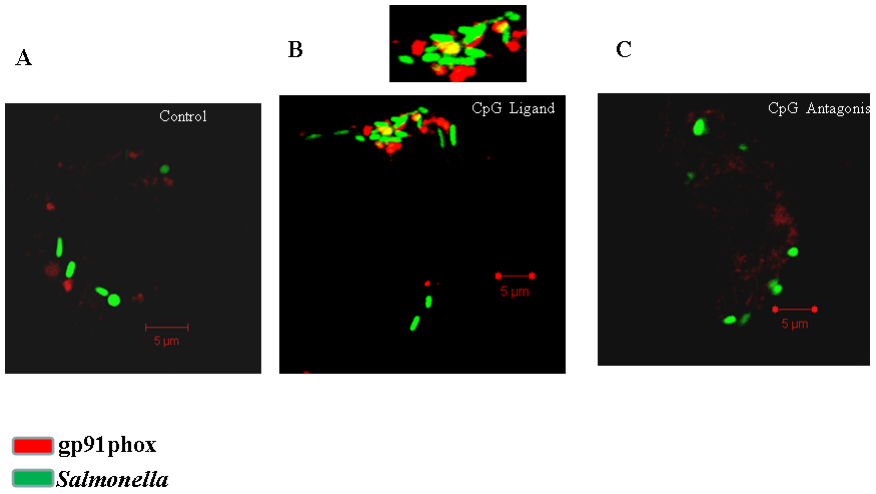
Localization of NADPH subunit gp91phox with the WT *Salmonella* using confocal microscopy. DC were infected with the WT-GFP for 12 h and stained with goat anti-mouse gp91phox IgG rabbit antibody followed by anti-goat IgG antibody conjugated to Cy3 (red). Representative pictures are shown from control cells (A), CpG treated cells (B) and antagonist treated cells (C).

In resting DCs the ROS generating complex is disassembled and inactive. However, in mature DCs in response to various stimulations, the cytosolic (p47phox, p67phox, p40phox and Rac2) and the membrane (gp91^phox^ and gp22^phox^) components are assembled together to form a functional enzyme and release oxygen radicals [25]–[27]. As it was evident that ROS production in the CpG treated DCs was significantly higher than the control cells, we next examined whether there is any difference in the NADPH phagocytic oxidase subunit localization between the two conditions. After infecting DC with the WT-GFP bacteria for 12 h, cells were stained for gp91phox subunit of NADPH phagocytic oxidase. Interestingly, increased co-localization of the WT and gp91phox was observed in the CpG treated cell (Fig. 5B) as compared to the control (Fig. 5A). In the ligand treated cells, the gp91 phox or the ROS complex staining was distinctly located near the bacteria containing regions. There were enhanced colocalization with the bacteria and also the ROS complex was found to be in close viscinity to the bacteria. This difference in the distribution and colocalization of the complex itself suggests that CpG treatment leads to direct bacterial killing by inducing ROS and not by any other mechanism. Antagonist treatment produced similar staining pattern like the control cells (Fig. 5C). When various fields were counted, the percentage co-localization of gp91phox with the WT was 4% in the control DCs in contrast to an enhanced 19% in the CpG treated DCs (data not shown).

### CpG mediated TLR-9 activation increases the capacity of DCs to process and/present *Salmonella* antigen in a ROS dependent manner

**Figure 6:**
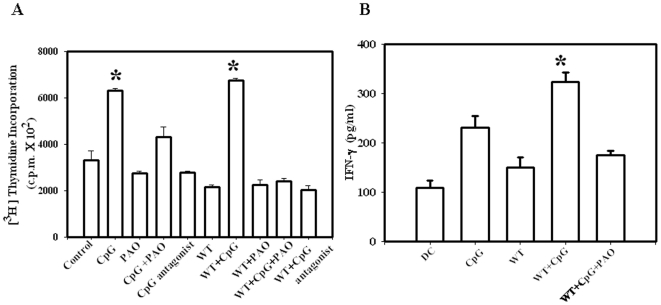
CpG DNA mediated TLR-9 activation leads to an enhanced *Salmonella* antigen presentation as measured by MLR. (A) Dendritic cells infected with the WT *Salmonella* were treated with 10 µg/ml CpG DNA or 10 µg/ml TLR-9 antagonist or 5 µM PAO. After 12 h post infection the total T cells isolated from the spleen of allogenic mice were added to each well. DC and T cell ratio was maintained at 1∶10. Cells were cocultured for 72 hr and then pulsed for 16 hr with [^3^H] thymidine (0.1 µCi/well). [^3^H] thymidine incorporation was measured in a liquid scintillation counter as mean counts/min of triplicate wells. (B) IFN-γ production from the same cell supernatant was measured by ELISA and the values were plotted as pg/ml of IFN-γ produced. Experiments were performed in triplicate on three different occasions and the standard errors from the means are shown. The statistics is defined as * *P*<0.05 (Student's *t* test).

There are contradictory reports of the effect of *Salmonella* infection in the antigen presentation by the dendritic cells. It has been previously reported that intracellular *Salmonella* affect the antigen presentation capacity of DCs. *Salmonella* infection in DCs inhibit T cell proliferation in a *Salmonella* pathogenicity island 2 and NO dependent manner. Interestingly, a recent report suggests that if *Salmonella* antigen is carried to the DCs by extracellular vesicles from infected macrophages a specific T cell response is generated [28]. In addition, injection of *Salmonella*-loaded DCs into mice result in both the CD4^+^ and CD8^+^ T cell activation specific for the model antigen used [29]. Thus, to better understand the effect of CpG on *Salmonella* antigen presentation in DCs, we performed mixed lymphocyte reaction (MLR). Our data revealed that *Salmonella* infected DCs from Balb/c mice do not effectively present antigen to the allogenic mixed T cell population from C57BL/6 mice spleen (Fig. 6A). There is actually a drop in the T cell proliferation in the *Salmonella* treated cells (Bar 6) than even the control cells (Bar 1). However, in the CpG DNA treated condition the antigen presentation and the T cell proliferation of the *Salmonella* infected DCs was significantly increased. It is also observed that only CpG treated cells (Bar 2) showed high antigen presentation. This finding indicates wild type *Salmonella* could inhibit antigen presentation from the infected DCs, but not from the CpG treated cells. TLR-9 antagonist treated DCs showed no difference in antigen presentation. We next substantiated our finding by measuring the IFN-γ production of the proliferating T cells. The T cells added to *Salmonella* infected DCs along with TLR-9 ligand treatment produced significantly higher amount of IFN-γ than without treatment (Fig. 6B). Since it is well known that ROS production affects antigen presentation in DCs we examined the effect of PAO during *Salmonella* infection. PAO treatment has no other adverse effect on DC death/survival (Figure S1) or maturation (Figure S2). Under PAO treatment (Bar 9), the CpG DNA mediated enhanced antigen presentation was no longer observed. Hence, it can be concluded that the CpG mediated enhanced ROS production allows better presentation of *Salmonella* antigen like any other antigen.

### TLR-9 activation enhances the capacity of DCs to process and/present exogenous antigen in a ROS dependent manner

**Figure 7:**
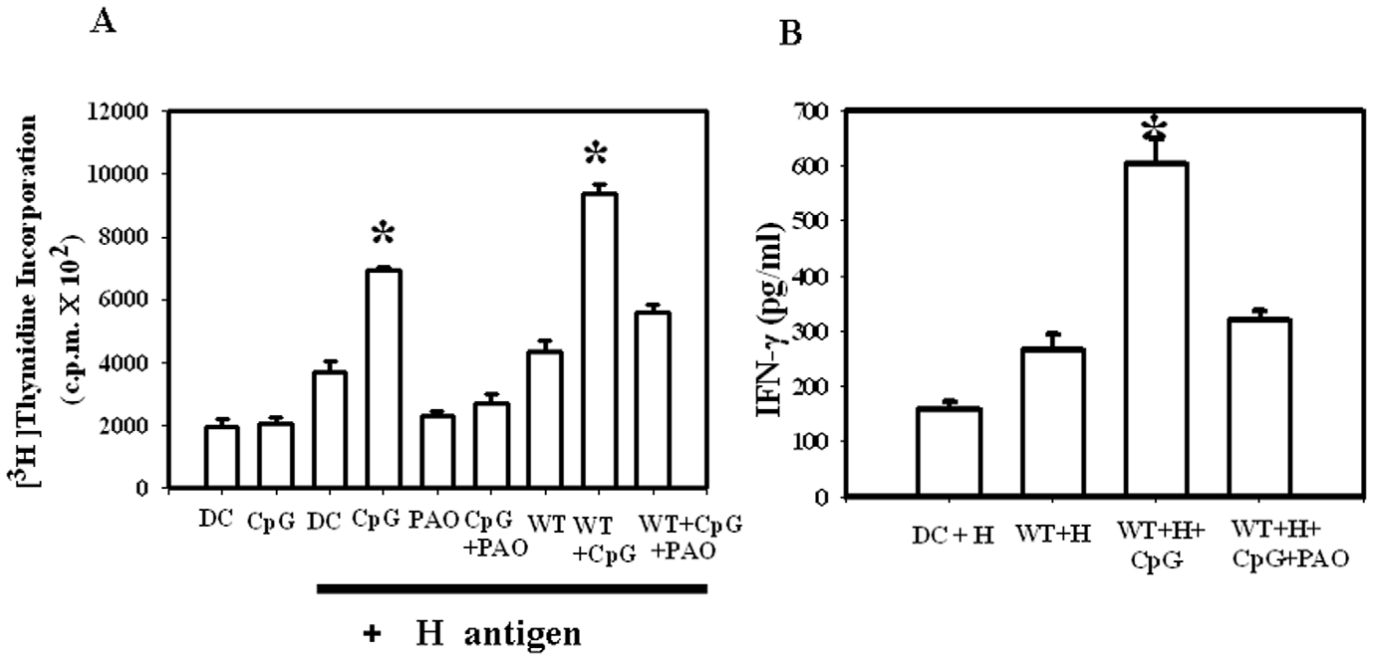
CpG DNA mediated TLR-9 activation leads to an enhanced *Salmonella* antigen presentation as measured by the H antigen presentation assay. (A) Dendritic cells were infected with the WT *Salmonella* along with the H antigen. 10 µg/ml CpG DNA or 10 µg/ml TLR-9 antagonist or 5 µM PAO were added to the DCs along with 10 µg/ml gentamicin. 12 h post infection, previously purified specific anti-H CD8^+^ T cells were added to each well. DC and T cell ratio was maintained at 1∶10. Cells were cocultured for 72 hr and then pulsed for 12 hr with [^3^H] thymidine (0.1 µCi/well). [^3^H] thymidine incorporation was measured in a liquid scintillation counter as mean counts/min of triplicate wells. (B) IFN-γ production from the same cell supernatant was measured by ELISA and the values were plotted as pg/ml of IFN-γ produced. Experiments were performed in triplicate on three different occasions and the standard errors from the means are shown. The statistics is defined as * *P*<0.05 (Student's *t* test).

We further questioned whether the enhanced antigen presentation occurs with a purified exogenous antigen as well. For this purpose, we used the purified envelope glycoprotein haemagglutinin (H-antigen) of rinder-pest virus and specific CD8^+^ T cells that were generated earlier from H-2d restricted mice [30], [31]. The process by which exogenous antigen get processed by the MHC I pathway and are presented to the CD8^+^ T cells is known as cross-presentation [32]. Antigens from intracellular pathogens like *Salmonella* have been shown to elicit a MHC class I dependent CD8^+^ T cell response and this process is also termed as cross-presentation. There are various explanations for cross-presentation. However, it has been convincingly shown that phagosomes are self sufficient organelles for the cross-presentation of any exogenous antigen [33]. In addition, it has also been reported that in the dendritic cells, endoplasmic reticulum and phagosome fusion leads to the generation of a specialized compartment capable of cross-presentation [8]. It is further known that TLR-3 and TLR-9 ligands induce cross-presentation in the dendritic cells [34]. Therefore, we asked whether CpG mediated TLR-9 activation could alter the cross-presentation capacity of the *Salmonella* infected DCs. Thus, we examined whether the exogenous antigen is taken up by the DCs and get presented MHC-I pathway. For this purpose, we treated the infected DCs with H-antigen and checked for the specific CD8^+^ T cell proliferation. In these sets of experiments a different trend of result was observed when compared to our MLR assay. Fig. 7A clearly indicates that H antigen cross-presentation gets significantly enhanced upon CpG DNA treatment. Although, *Salmonella* infection (Bar 7) did not enhance the antigen presentation capacity of the control cells (Bar 3), we did not observe a decrease in the antigen presentation after *Salmonella* infection like the MLR assay. However, interestingly we noticed that the H-antigen cross presentation is increased in the infected CpG treated cells (Bar 8). There was an enhanced antigen presentation in the control cells treated with CpG (Bar 4) but after *Salmonella* infection this was further enhanced. *Salmonella* could not downregulate the enhanced antigen presentation. Blocking the ROS production with PAO prevented this enhanced cross presentation clearly indicating the fact that the enhanced antigen presentation is actually mediated by CpG induced ROS production. These results indicate that if we use CpG it might lead to enhanced presentation of *Salmonella* antigen also. However, to confirm this finding, we need to perform the experiment with an assay system to detect specific *Salmonella* antigen cross-presentation. Further, analysis of the cytokine profile showed a positive correlation with the T cell proliferation assay. As shown in Fig. 7B, the IFN-γ production was significantly low in case of only *Salmonella* infection without CpG DNA treatment. In case of *Salmonella* infection with CpG DNA treatment anti-H T cells produced significantly higher amount of IFN-γ. This enhanced IFN-γ production was abrogated with PAO treatment.

### Lower burden of *Salmonella* in the organs of CpG induced DC treated mice

**Figure 8:**
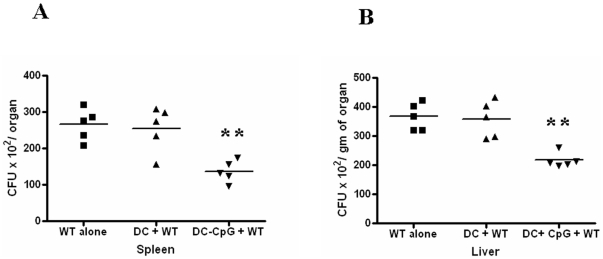
‘CpG treated DC’ transfer in mice followed by *Salmonella* infection resulted in reduced bacterial proliferation in vivo. Mice were treated intraperitoneally with either DC alone or ‘CpG treated DC’. Control mice were treated with PBS. After 7 days of treatment, 1×10^6^ s of the WT strain were inoculated orally to the mice groups. After 5^th^ day of infection, homogenized samples of (A) spleen and (B) liver of the infected mice were plated on antibiotic plates and the colonies were counted. Result presented is one of two independent experiments. Statistical significance was defined as follows: **, P<0.01 (Mann Whitney U test).

DCs are the principal cells wherein *Salmonella* reside after host invasion in the sub epithelial dome of the Peyer's patches [35]. In fact, *Salmonella* use DCs as a means of infecting M cells and employ the high infiltration capacity of the dendritic cells to help in its dissemination to other organs [29], [36]. To extrapolate our findings in an in vivo context, we intraperitoneally injected control DCs or CpG treated DCs to mice and after 7 days infected them with WT *Salmonella*. We analyzed the bacterial load after 5 days in the spleen and liver after infecting mice orally at a dose of 10^6^ bacteria per mouse. Data presented in Fig. 8A-B clearly indicates that there was a significant decrease in the bacterial burden in all the organs of mice treated with the CpG induced DCs when compared to the mice treated with control DCs or PBS treated mice.

## Discussion

We observed that CpG DNA treatment inhibits *Salmonella* Typhimurium growth in murine DCs in a ROS dependent manner. Further, our study unravels the role of ROS in promoting antigen presentation in *Salmonella* infected DCs. An enhanced antigen presentation was seen in the *Salmonella* infected dendritic cells upon CpG treatment. *Salmonella* infected cells showed a decrease in the allogenic T cell proliferation than the control cells. However, *Salmonella* could not decrease the CpG induced enhanced T cell proliferation. Hence, it can be concluded that *Salmonella* utilizes the unique strategy of inhibiting the DC mediated antigen presentation. However, activating the host innate receptor TLR-9 allows an efficient presentation of *Salmonella*. Further, CpG treated DCs when injected in the mice system could reduce the *Salmonella* load from various organs suggesting this result can be extrapolated in vivo as well. Thus, our data identifies a strategy to increase the efficiency of antigen presentation by *Salmonella* infected DCs.

The intracellular life of *Salmonella* in the DC is very interesting. *Salmonella* does not get killed or proliferate in the DC population. Intracellular survival of *S.* Typhimurium in murine DC was independent from the function of virulence factors known to be important for survival in macrophages, such as the SPI2-T3SS. Surprisingly, the bacterial LPS O antigen is found to be very crucial to maintain bacterial steady burden in the DCs [37]. As known from previous studies, the effect of CpG mediated TLR-9 activation on *Salmonella* proliferation is a cell line dependent phenomenon. In the case of cultured hepatocytes, *Salmonella* growth is inhibited by the CpG DNA treatment [23], whereas in the case of human monocyte-derived macrophages [38] it has no effect. In hepatocytes, an enhanced ROS production after the CpG DNA treatment has been attributed to be the cause of decreased *Salmonella* burden [23]. However, in bone marrow derived macrophages CpG-antagonist act in a TLR-9 independent manner to inhibit *Salmonella* killing [39]. Our study is the first report which indicates that in the dendritic cells CpG DNA enhances *Salmonella* killing. We went ahead to check the exact mechanism behind, and noticed that CpG induction leads to an enhanced generation of ROS from the infected cells. To look into the mechanism behind this phenomenon, we blocked the ROS production in the ligand treated infected dendritic cells. Under that condition this enhanced killing was no more observed. This finding clearly demonstrates that the CpG treatment allows better killing of *Salmonella* in a ROS dependent manner.

Several report have documented that *Salmonella* inhibits antigen presentation from DC to MHC class II molecules. This effect was dependent on the induction of inducible NO synthase by DC and on the function of virulence genes in SPI2, as SPI2 deficient bacterial infection led to an enhanced amount of both CD4 and CD8 cells [40]. SPI-2 encoded proteins like SifA, SspH2, SlrP, PipB2, and SopD2 were found to be important for the interference with antigen presentation, whereas SseF and SseG contributed to a lesser extent to this phenotype [41]. Published data clearly outline the pivotal role of ROS in the antigen presenting cells [20]. Maintaining the redox potential is extremely important for macrophages and DCs to present antigen in the context of MHC molecules [16]. Oxidative stress has been reported to modulate the signal transduction pathways in lymphocytes [17]. It has been further observed that antioxidants like NAC, quercetin and polyphenolic compounds directly inhibit B cell mediated antigen presentation by inhibiting NFκb activation [19]. Moreover, the antigen specific DC-T cell interaction can be specifically blocked by modulating the redox potential of DCs [42]. These findings led us to look into the antigen presentation capacity of the CpG treated infected cells as the cells were producing higher amount of ROS. So, we went ahead to measure the antigen presentation by the help of a mixed lymphocytic reaction. The role of CpG DNA has been well documented as an adjuvant in multiple experimental and clinical vaccines [43]. Therefore, our study indicates that CpG DNA can be used as an adjuvant to design new vaccines with increased efficiency against *Salmonella*. Our next aim was to check the role of CpG in aiding cross presentation in the *Salmonella* infected DC. We observed that unlike MLR assay *Salmonella* does not downregulate CD8 T cell proliferation than the control cells which was also observed by other groups previously [44]. However, CpG treatment leads to enhanced cross presentation from the DC and this was also dependent on the ROS production. When we allowed *Salmonella* infection in the CpG treated DCs, the same trend was maintained. *Salmonella* could not alter the enhanced antigen presentation and thus we hypothesize that like H-antigen any other *Salmonella* antigen could also be cross-presented in a better way by CpG induction. This idea is further supported by the animal experiments performed with the CpG treated DCs. The injected DCs could reduce the bacterial load from infected mice from both liver and spleen. This might be due to enhanced antigen presentation and killing of the bacteria by the anti-*Salmonella* T cells.

To be precise, this paper deals with two novel findings. In one hand, CpG treatment leads to killing of *Salmonella* in a ROS dependent manner. On the other, CpG induction leads to better antigen presentation from the *Salmonella* infected DCs which is also dependent on ROS. These two findings might be related in a way as enhanced killing of the pathogen might allow better presentation as well which is again supported by the *in vivo* data. In conclusion, our finding is the first to indicate that activating host innate immune receptor TLR-9 can improve *Salmonella* killing and antigen presentation by DCs. We speculate that our results will have important implications in the development of novel *Salmonella* vaccines utilizing CpG as an adjuvant. Future studies should focus on the exact mechanism by which ROS enhances the antigen presentation in the *Salmonella* infected DCs.

## Materials and Methods

### Ethics Statement

All the work with animals has been done with Institution approved ethics protocol. The ethics number being CAF/ETHICS/189/2010.

### Preparation of dendritic cell cultures

Murine bone marrow derived dendritic cells were cultured from the Balb/C mice as described before [40]. Briefly, the femurs were collected aseptically from mice and the marrow was flushed out. The cells were spun at 1,000 rpm for 10 min and resuspended in RPMI containing 10 ng/ml GMCSF (Peprotech), 10% heat-inactivated FBS, 2 mM l-glutamine, 100 U/ml penicillin, 100 µg/ml streptomycin and plated in 75-cm^2^ tissue culture flasks. The flasks were placed in a tissue culture incubator in 10% CO_2_ at 37°C and after 3 days GMCSF supplementation was done. Finally, after 6 days of culture the medium was removed, and the cells were washed once and detached by pipetting. The cells were observed to be >90% CD11c positive by fluorescence-activated cell sorting analysis. The above mentioned cell suspension was further purified using CD11c^+^ magnetic beads (MACS, Miltenyi Biotec). The purified DCs were plated at 1×10^6^ cells per ml in 6-well tissue culture plates 6 h prior to use.

### Cell culture and bacterial infection

For the infection of cells, the wild type (WT) *Salmonella* Typhimurium strain was grown to stationary phase in LB in the presence of carbenicillin. The OD (600) of the culture was adjusted with Luria broth (LB) to 0.3 and the bacteria were washed once with phosphate-buffered saline (PBS). Appropriate dilution of the bacterial culture was made in cell culture medium and added to the dendritic cells growing in 24-well tissue culture plates at a multiplicity of infection (MOI) of about 10. Bacteria were centrifuged onto the cells at 500×g for 5 min. After infection for 25 min, cells were washed thrice with PBS and incubated for 1 h in cell culture medium containing 50 µg/ml gentamicin (Sigma). The cells were maintained in 10 µg/ml gentamicin containing medium for the rest of the experiment. In some sets of the experiment, 10 µg/ml TLR-9 ligand (CpG-ODN-1826, Invivogen) or 10 µg/ml TLR-9 antagonist (CpG-ODN-2088, Invivogen) was added to the cells along with 10 µg/ml gentamicin. In some experiments, cells were pretreated for 30 min with PAO (phenylarsine oxide, 5 µM) before performing the infection. For the enumeration of the intracellular bacteria, the DCs were washed three times with PBS and lysed with 0.1% Triton X-100 for 10 min at room temperature and serial dilutions were plated onto LB agar plates containing carbenicillin.

### Western blot analysis

In order to detect TLR-9, aliquots containing equal amounts of protein (100 µg) were loaded onto 12.5% gel and transferred onto nitrocellulose membranes using mini gel transfer apparatus (Bio-Rad). The membranes were incubated with mouse monoclonal antibody against mouse TLR-9 (BD transduction laboratories; 1∶500) for 2 h. The blots were further treated with goat anti mouse IgG-horseradish peroxidase conjugate (AP-Biotech, dilution 1∶2000) for 2 h. The immune complex on the blots were detected with enhanced chemiluminescence substrate (PerkinElmer) and exposed to Eastman Kodak Co. XAR x-ray film. All blots were stripped and stained with HRP conjugated anti β-actin antibody (Sigma, 1∶1000).

### Measurement of intracellular ROS

Intracellular ROS was measured by flow cytometry in cells using the redox-sensitive dye 2′, 7′-dichlorodihydrofluorescein diacetate (H_2_DCFDA). H_2_DCFDA (Molecular probes) is a cell-permeant indicator for ROS that is nonfluorescent until the acetate groups are removed by intracellular esterases and it is oxidised within the cell. H_2_DCFDA readily diffuses into the cells wherein it is oxidised to the highly fluorescent polar derivative H_2_DCF in the presence of H_2_O_2_. 1×10^6^ cells were incubated for 30 min with 10 µM H_2_DCFDA in dark. Cells were quickly washed and immediately 10,000 events were acquired on a FACSCalibur (BD Biosciences).

### Localization of NADPH subunit, gp91phox by fluorescence microscopy

Localization of gp91phox in the DCs were determined by standard immunocytochemical methods. The WT-GFP infected cells were washed free of medium and the cells were fixed for 10 min in paraformaldehyde (3.5%) at room temperature and washed with PBS. Fixed cells were then incubated with goat anti-mouse gp91phox immunoglobulin G (BD Transduction) diluted 1∶100 in PBS containing 2%BSA, 2% goat serum and 0. 1% saponin. The cells were then washed three times in PBS and incubated in identical conditions but with rabbit anti-goat IgG conjugated to Cy3 (Jackson's lab). Samples were viewed on a confocal laser-scanning microscope equipped with an argon laser (Zeiss, Germany). For determining the % co-localization, 50 microscopic fields were counted.

### Splenocyte preparation

Total splenocytes were prepared from C57BL/6 mice by incubating small pieces of spleens in serum-free, calcium-free HBSS at 37°C for 45 min. This preparation was disaggregated by pipetting to produce a single-cell suspension. The cells were then washed in HBSS, and erythrocytes were lysed by hypotonic shock. Preparations were filtered to remove debris and were resuspended in RPMI.

### Mixed lymphocyte reaction

Dendritic cells were isolated from the Balb/C mice as described earlier and were plated at a density of 5×10^5^ cells/well in 96-well flat-bottomed tissue culture plates. Bacterial infections of the DCs were performed as described previously. 10 µg/ml TLR-9 ligand, 10 µg/ml TLR-9 antagonist or PAO (5 µM, phenylarsine oxide) were added to the cells along with 10 µg/ml gentamicin. After 12 h post infection, total T cells isolated from spleens of allogenic C57BL/6 mice (as described above) were added to each well. DC and T cell ratio was maintained at 1∶10. Cells were cocultured for 72 hr and then pulsed for 16 hr with [^3^H] thymidine (0.1 µCi/well). Cells were harvested on glass fibre filters using a semi-automated cell harvester (Nunc, Roskilde, Denmark). [^3^H] thymidine was measured in a liquid scintillation spectrophotometer and T-cell proliferation was measured as mean counts/min of triplicate wells.

### H antigen presentation assay

Dendritic cells were isolated as described earlier and were plated at a density of 5×10^5^ cells/well in 96-well flat-bottomed tissue culture plates. Bacterial infection of the DCs was performed as described previously. A soluble form of previously [31] purified envelope glycoprotein haemagglutinin (H-antigen) of rinder-pest virus (50 µg/ml) was added to the cells along with 10 µg/ml gentamicin. After 12 h post infection responder T cells (specific CD8^+^ T cells that were generated earlier from H-protein-immunized mice) were added to each well and a proliferation assay was carried out as described for the mixed lymphocytic reaction. The specific CD8 T cell clone was generated earlier. Briefly, inguinal lymph nodes from H immune mice were collected one week after booster immunization and co-cultured with irradiated syngenic normal mouse splenocytes in 1.5 ml complete medium containing 50 µg/ml H protein. To obtain T cell clones, limiting dilution of cultured cells was performed in presence of irradiated syngenic splenocytes and IL-2. Single cells from a growing colony were selected and expanded for testing antigen specificity and surface markers. To test the antigen specificity and MHC restriction of T cell clones, cells were co-cultured with irradiated P815 cells stably expressing H protein and with or without purified anti-mouse Kd antibody (e-Bioscience, USA). Generated H specific T cell clone on staining for surface CD marker was found to be CD8^+^. The antigen specificity of the T cell clone was established using these cells as effectors cells in a CTL assay.

### Cytokine ELISA

Splenocytes from C57BL/6 mice were incubated with *Salmonella* infected DCs from the Balb/C mice for 72 hr as described above for cell proliferation assay. Supernatants from the culture were collected for cytokine assays. Interferon-γ (IFNγ) was assayed using cytokine ELISA kits (BD-Bioscience).

### Animals

Six to eight-week-old Balb/C and C57BL/6 mice were maintained under specific-pathogen-free conditions in Central Animal Facility, Indian Institute of Science, Bangalore, India. All the procedures with animals were carried out as approved by the animal ethic's committee of the Institute.

### *In vivo* transfer of DCs and organ load

Female BALB/c (n = 5) were intra peritoneally injected with 10^8^ DCs (untreated or CpG treated) dissolved in 100 µl PBS. After 7 days of treatment, the mice were orally infected with 10^6^ CFU of the WT strain. After 5^th^ day of infection, homogenized samples of liver, spleen and MLN of infected mice were plated on antibiotic plates and the colonies were counted.

### Statistical analysis and software

Each assay was repeated at least 3 times. In vitro data were analyzed by paired *t* test (two sample, equal variance) and P values below 0.05 were considered significant. FACS data were plotted and analyzed using WinMDI 2.9 software. Results of mouse *in vivo* challenge studies were evaluated by using Mann-Whitney U tests from the GraphPad Prism 4.0 software. Immunoblots were quantified using Multi Gauge V2.3 software. Differences between experimental groups were considered significant for *P*<0.05.

## Supporting Information

Figure S1 MTT assay. DCs were infected in triplicate with the WT strain with/without different treatments. After 12 h of infection, MTT assay was performed and the values are represented as % cell viability considering the uninfected cell viability as 100%. There was no significant cell death in any of the conditions tested.
(0.06 MB TIF)

Figure S2 Activation assay. DCs were infected in triplicate with the WT strain with/without different treatments. After 12 h of infection, FACS was performed for the dendritic cell maturation marker CD80 and the maturation statuses of the DCs are shown as MFI. Only CpG treatment leads to an enhanced maturation of the DCs. The statistics is defined as * P<0.05 (Student ‘t’ test).
(0.06 MB TIF)

## References

[1] Reis e Sousa C (2001). Dendritic cells as sensors of infection.. Immunity.

[2] Riezman H, Woodman PG, van Meer G, Marsh M (1997). Molecular mechanisms of endocytosis.. Cell.

[3] Xiang SD, Scholzen A, Minigo G, David C, Apostolopoulos V (2006). Pathogen recognition and development of particulate vaccines: does size matter?. Methods.

[4] Lanzavecchia A, Sallusto F (2001). Regulation of T cell immunity by dendritic cells.. Cell.

[5] Guermonprez P, Valladeau J, Zitvogel L, Thery C, Amigorena S (2002). Antigen presentation and T cell stimulation by dendritic cells.. Annu Rev Immunol.

[6] Savina A, Amigorena S (2007). Phagocytosis and antigen presentation in dendritic cells.. Immunol Rev.

[7] Banchereau J, Briere F, Caux C, Davoust J, Lebecque S (2000). Immunobiology of dendritic cells.. Annu Rev Immunol.

[8] Guermonprez P, Saveanu L, Kleijmeer M, Davoust J, Van Endert P (2003). ER-phagosome fusion defines an MHC class I cross-presentation compartment in dendritic cells.. Nature.

[9] Kaisho T, Akira S (2004). Pleiotropic function of Toll-like receptors.. Microbes Infect.

[10] Miggin SM, O'Neill LA (2006). New insights into the regulation of TLR signaling.. J Leukoc Biol.

[11] Kawai T, Akira S (2007). TLR signaling.. Semin Immunol.

[12] Blander JM (2007). Coupling Toll-like receptor signaling with phagocytosis: potentiation of antigen presentation.. Trends Immunol.

[13] Watson JL, McKay DM (2006). The immunophysiological impact of bacterial CpG DNA on the gut.. Clin Chim Acta.

[14] Babior BM, Lambeth JD, Nauseef W (2002). The neutrophil NADPH oxidase.. Arch Biochem Biophys.

[15] McPhail LC, Strum SL, Leone PA, Sozzani S (1992). The neutrophil respiratory burst mechanism.. Immunol Ser.

[16] Mantegazza AR, Savina A, Vermeulen M, Perez L, Geffner J (2008). NADPH oxidase controls phagosomal pH and antigen cross-presentation in human dendritic cells.. Blood.

[17] Nakamura H, Nakamura K, Yodoi J (1997). Redox regulation of cellular activation.. Annu Rev Immunol.

[18] Turpaev KT (2002). Reactive oxygen species and regulation of gene expression.. Biochemistry (Mosc).

[19] Gong J, Chen SS (2003). Polyphenolic antioxidants inhibit peptide presentation by antigen-presenting cells.. Int Immunopharmacol.

[20] Savina A, Jancic C, Hugues S, Guermonprez P, Vargas P (2006). NOX2 controls phagosomal pH to regulate antigen processing during crosspresentation by dendritic cells.. Cell.

[21] Maemura K, Zheng Q, Wada T, Ozaki M, Takao S (2005). Reactive oxygen species are essential mediators in antigen presentation by Kupffer cells.. Immunol Cell Biol.

[22] Latz E, Verma A, Visintin A, Gong M, Sirois CM (2007). Ligand-induced conformational changes allosterically activate Toll-like receptor 9.. Nat Immunol.

[23] Sanchez-Campillo M, Chicano A, Torio A, Martin-Orozco E, Gamiz P (2004). Implication of CpG-ODN and reactive oxygen species in the inhibition of intracellular growth of Salmonella typhimurium in hepatocytes.. Microbes Infect.

[24] Ewaschuk JB, Backer JL, Churchill TA, Obermeier F, Krause DO (2007). Surface expression of Toll-like receptor 9 is upregulated on intestinal epithelial cells in response to pathogenic bacterial DNA.. Infect Immun.

[25] McPhail LC (1994). SH3-dependent assembly of the phagocyte NADPH oxidase.. J Exp Med.

[26] Vignais PV (2002). The superoxide-generating NADPH oxidase: structural aspects and activation mechanism.. Cell Mol Life Sci.

[27] Vulcano M, Dusi S, Lissandrini D, Badolato R, Mazzi P (2004). Toll receptor-mediated regulation of NADPH oxidase in human dendritic cells.. J Immunol.

[28] Eguchi M, Sekiya Y, Kikuchi Y, Takaya A, Yamamoto T (2007). Expressed Salmonella antigens within macrophages enhance the proliferation of CD4+ and CD8+ T lymphocytes by means of bystander dendritic cells.. FEMS Immunol Med Microbiol.

[29] Yrlid U, Svensson M, Johansson C, Wick MJ (2000). Salmonella infection of bone marrow-derived macrophages and dendritic cells: influence on antigen presentation and initiating an immune response.. FEMS Immunol Med Microbiol.

[30] Vani J, Nayak R, Shaila MS (2007). Maintenance of antigen-specific immunological memory through variable regions of heavy and light chains of anti-idiotypic antibody.. Immunology.

[31] Vani J, Nayak R, Shaila MS (2007). A CD8+ T cell clone specific for antigen also recognizes peptidomimics present in anti-idiotypic antibody: implications for T cell memory.. Cell Immunol.

[32] Rock KL (2003). The ins and outs of cross-presentation.. Nat Immunol.

[33] Houde M, Bertholet S, Gagnon E, Brunet S, Goyette G (2003). Phagosomes are competent organelles for antigen cross-presentation.. Nature.

[34] Datta SK, Redecke V, Prilliman KR, Takabayashi K, Corr M (2003). A subset of Toll-like receptor ligands induces cross-presentation by bone marrow-derived dendritic cells.. J Immunol.

[35] Kirby AC, Yrlid U, Svensson M, Wick MJ (2001). Differential involvement of dendritic cell subsets during acute Salmonella infection.. J Immunol.

[36] Yrlid U, Svensson M, Kirby A, Wick MJ (2001). Antigen-presenting cells and anti-Salmonella immunity.. Microbes Infect.

[37] Zenk SF, Jantsch J, Hensel M (2009). Role of Salmonella enterica lipopolysaccharide in activation of dendritic cell functions and bacterial containment.. J Immunol.

[38] Wang JP, Hayashi T, Datta SK, Kornbluth RS, Raz E (2005). CpG oligonucleotides partially inhibit growth of Mycobacterium tuberculosis, but not Salmonella or Listeria, in human monocyte-derived macrophages.. FEMS Immunol Med Microbiol.

[39] Trieu A, Bokil N, Dunn JA, Roberts TL, Xu D (2009). TLR9-independent effects of inhibitory oligonucleotides on macrophage responses to S. typhimurium.. Immunol Cell Biol.

[40] Cheminay C, Mohlenbrink A, Hensel M (2005). Intracellular Salmonella inhibit antigen presentation by dendritic cells.. J Immunol.

[41] Halici S, Zenk SF, Jantsch J, Hensel M (2008). Functional analysis of the Salmonella pathogenicity island 2-mediated inhibition of antigen presentation in dendritic cells.. Infect Immun.

[42] Matsue H, Edelbaum D, Shalhevet D, Mizumoto N, Yang C (2003). Generation and function of reactive oxygen species in dendritic cells during antigen presentation.. J Immunol.

[43] Lahiri A, Das P, Chakravortty D (2008). Engagement of TLR signaling as adjuvant: towards smarter vaccine and beyond.. Vaccine.

[44] Lapaque N, Hutchinson JL, Jones DC, Meresse S, Holden DW (2009). Salmonella regulates polyubiquitination and surface expression of MHC class II antigens.. Proc Natl Acad Sci U S A.

