# Supplementary figures and images for: TLR 9 Activation in Dendritic Cells Enhances Salmonella Killing and Antigen Presentation via Involvement of the Reactive Oxygen Species
Source: PLoS One. 2010 Oct 29;5(10):e13772. doi: 10.1371/journal.pone.0013772 (PMC2966436; doi:10.1371/journal.pone.0013772)

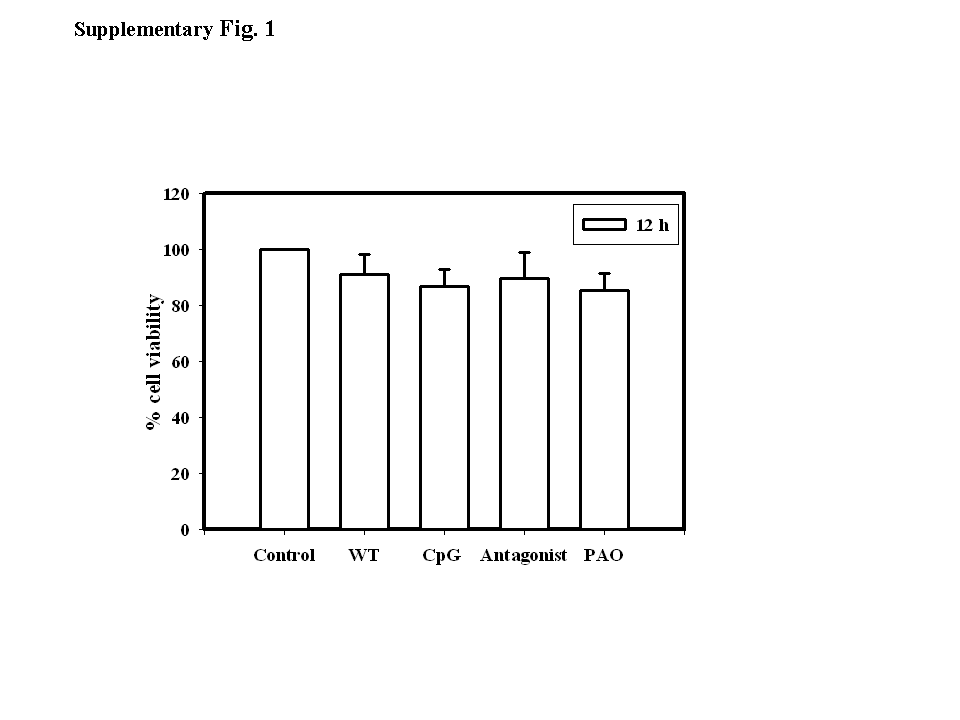

Supplement: Figure S1 — MTT assay. DCs were infected in triplicate with the WT strain with/without different treatments. After 12 h of infection, MTT assay was performed and the values are represented as % cell viability considering the uninfected cell viability as 100%. There was no significant cell death in any of the conditions tested. (0.06 MB TIF) [file pone.0013772.s001.tif]

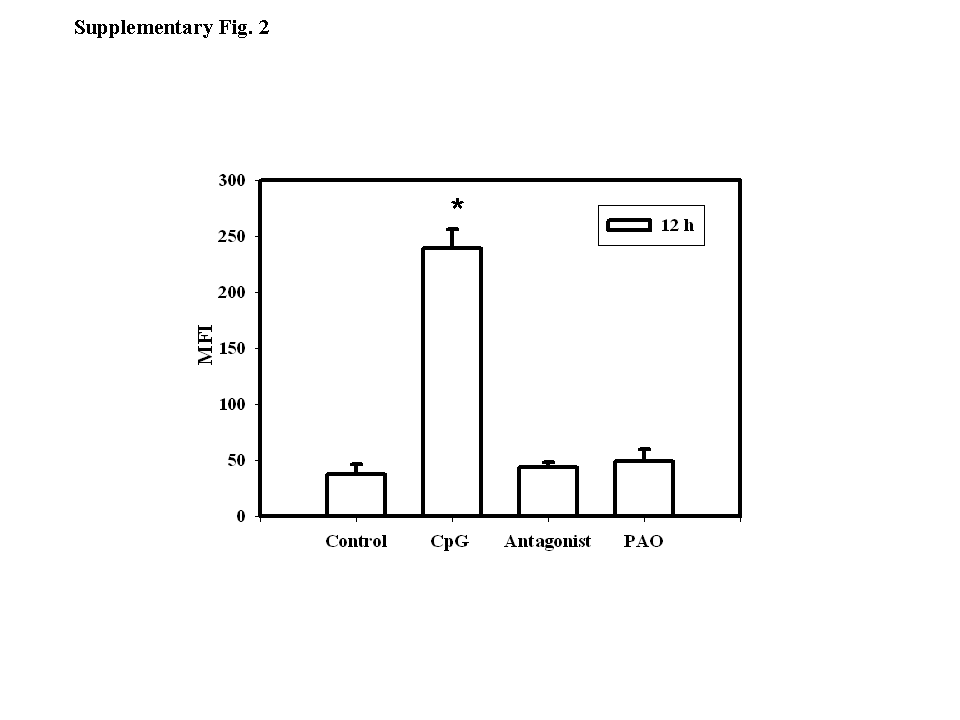

Supplement: Figure S2 — Activation assay. DCs were infected in triplicate with the WT strain with/without different treatments. After 12 h of infection, FACS was performed for the dendritic cell maturation marker CD80 and the maturation statuses of the DCs are shown as MFI. Only CpG treatment leads to an enhanced maturation of the DCs. The statistics is defined as * P<0.05 (Student ‘t’ test). (0.06 MB TIF) [file pone.0013772.s002.tif]
